# Morbid Effects of Tight-Lacing

**Published:** 1846-08

**Authors:** Thos. W. Carter


*The Morbid Effects of Tight-Lacing.* By Thos. W. Carter, M. D.—The author of this article, which we find in the Southern Medical and Surgical Journal for July, arrives at the following conclusions:

That the excessive prevalence of uterine derangements, such as the various displacements of the uterus, abortion, menorrhagia, amenorrhœa, leucorrhœa, &c., are attributable to tight-lacing; that the same practice induces constitutional debility in females, which is transmitted to the offspring when they become mothers.

Since I entered upon the duties of my profession, I have frequently been called on by elderly ladies to answer the following questions:—"What is the cause of the modern prevalency of uterine derangements, and more especially of prolapsus uteri? Why, in our youthful days, was it of rare occurrence, to meet with a woman thus affected—while of late it is equally rare to find one who is not at times more or less troubled with falling of the womb." Such questions naturally suggested to my mind, as the cause, some impropriety in the habits of the females of the present day. I knew it to be an unequivocal fact, that cases of prolapsus uteri and uterine derangements, generally, in modern times, compared with those of former days, (with due regard to increase of population,) were greatly in excess, if not in the ratio of three to one.

My being at first unable to give a decisive answer to these frequent and very rational inquiries, actuated me to inquire into the subject of the modern causes of the diseases under consideration, which brought me to the following conclusions: 1st. That the excessive prevalence of uterine derangements, in modern days, is owing to the unnatural and destructive practice of tight-lacing, so general among the female sex. 2nd. That such a practice does induce constitutional debility in the female economy. 3rd. That this constitutional debility is transmitted from the mother to her offspring, or at least the effects wrought upon the constitution of the mother affect her offspring.

I now propose examining each successively in the order in which they occur.

I. *That the excessive prevalence of uterine derangements, in modern times, is attributable to Tight-Lacing.*

In enumerating the uterine diseases which appear often to be engendered by this destructive fashion, we find the various displacements of the uterus—viz: prolapsus, procidentia, retroversion, &c., standing first in the catalogue. 2nd, abortion; 3rd, menorrhagia, 4th, the various derangements of the uterine functions—viz: amenorrhœa, dysmenorrhœa, leucorrhœa, &c., &c.

1st. *The various displacements of the Uterus.*—The manner in which tight-lacing is instrumental, in the poduction of these loathsome maladies, is plain and obvious. The cruel compression exerted by an unyielding bodice, environing the inferior portion of the thorax and the superior portion of the abdomen, cannot but act injuriously. It forces the contents of the abdominal upon those of the pelvic cavity. From the long-continued and almost constant intrusions of the abdominal viscera, the ligaments which support the uterus become relaxed and so much weakened as no longer to be capable of sustaining that organ, together with the weight of the whole contents of the abdomen thus forced down: consequently the uterus, by degrees, is forced lower down into the pelvic cavity, until prolapsus, or some other displacement, is the unavoidable result.

2d. *Abortion.*—Abortion may be produced by plethora of the uterine system, which, from the engorged state of the capillaries of the uterus, is adequately calculated to induce a separation of the ovum at any period of gestation. Such a condition of the uterus is known to be inimical to the existence and safe repose of the ovum in its cavity: consequently, under such circumstances, conception is not likely to take place, and if it do take place, is likely soon to be followed by a separation and expulsion of the germ. This condition of the uterus may be readily induced during gestation, (even in those who have previously enjoyed good health,) by the impediment offered to the free return of the blood to the heart, by compression necessarily employed in *tight-lacing*, as well as the mechanical pressure of the uterus upon the pelvic veins, when that organ is more compressed by the abdominal viscera.

Abortion may also be induced by an opposite condition of the uterine system to that above described—that of anæmia, relaxation, &c.

In ladies who have been tenacious in adhering to the modern fashions from early life, this condition of the system is of common occurrence. In them we find an ample evidence of the debilitating and relaxing influence of modern fashion on the female economy, and more especially upon the functions of the uterine system.

Abortion may likewise be induced by an "inability in the uterus to extend itself beyond a certain size." We can readily imagine this condition of the organ, in the victims of the modern laced-jacket.

3rd. *Menorrhagia.*—This hemorrhagic discharge may be either active or passive, or at least connected with a plethoric or hyperæmic condition of the uterus, or with a debilitated or anæmic state of the general system. The former condition may be the immediate result of the mechanical impediment offered (by excessive lacing) to the free passage of the blood to the centre of the circulatory system, thereby inducing an engorged condition of the capillaries of the uterus. The latter condition (that of anæmia) may be the mediate or secondary result of this impediment offered to the circulation. The plethoric condition may not in every instance result in hemorrhage, but, by inducing menstrual irregularities and derangements, the general system will become chlorotic, and so much debilitated, the uterine capillaries relaxed, and the whole mass of blood so much vitiated and attenuated, as to result in passive menorrhagia.

4th. *Amenorrhœa, Dysmenorrhœa, Leucorrhœa, &c., &c.*—That the many menstrual irregularities which appear to be so very prevalent of late years, are in a great degree owing to the infatuation of modern fashion, no one of observation and information will deny. It is true, there are other causes which are very efficient in the production and perpetuation of menstrual irregularities and derangements, but I think it will be conceded by the reflecting mind that many of those derangements are attributable to female dress, and that much of the suffering and inconvenience to which many females are subject might be obviated, if they would dispense with the laced-jacket.

II. *That excessive lacing does induce constitutional debility in females.*

The practice of tight-lacing, we have seen, is very efficient in the production of the uterine derangements, in which the general system of necessity must participate. The whole mass of circulating fluid appears to become deteriorated.

The female, notwithstanding all her native beauties and perfections of form and constitution, is now compelled to lie down upon the bed of affliction and deplore the loss of all her fascinating charms. Where is the rosy tint that once decorated these pale and blanched cheeks? Where is that sprightly, buoyant, benignant expression that once sat upon this dejected countenance? Where that symmetrical, that beautiful, lovely form? Alas! how changed. We now behold the pallid cheek, the lusterless eye, the distorted countenance, indicative of pain and suffering—the emaciated frame and incurvated body immaturely and untimely drooping into the grave, before half the days allotted to woman have passed away.

Perhaps it may be proper, while upon this division of our subject, to notice some of the evil effects of tight-lacing upon the various organs of the body, and the mnltifarious diseases that may result therefrom.

The lungs appear to suffer materially from this practice. Any person of observation may satisfy himself of the truth of this assertion by observing the motions of the chest during respiration. He will observe that it is impossible fully to inflate the lungs. The respiration is short and quick, inspiration is about half as long as expiration, and there is a considerable interval between expiration and inspiration. During this interval, the lungs are inactive and blood is passing through them, without the benefit of the oxygen of the atmosphere, and thus proceeds unoxygenized to the heart—from thence to be distributed to the various organs of the body throughout the general system.

May not such a perversion of the function of respiration and consequent unfitness of the blood to subserve the important purposes of nutrition, be considered a very efficient cause of the early developement and maturation of the tuberculous deposition in the lungs? May it not be regarded a prolific source of that irritable and cachectic condition of the system which appears to characterize the modern fashionable lady. The heart, the stomach, the liver, and indeed almost all the viscera of the thorax and abdomen, appear to be obnoxious to injury from the improper encroachments of the laced-jacket, by which are induced, directly and indirectly, a host of diseases known to depend upon a deteriorated quality of the circulating fluid, together with a perverted and deranged nutrition.

Lacing, when carried to excess, is unfavorable to longevity in a high degree, terminating life ere the span be half measured. Show me a matron, who has attained her three score years and ten, and I will show you a lady who was a stranger to fashion in her youthful days.

For the purpose of corroborating the position I have assumed, let us draw a contrast between the rustic maid, and the city belle, the victim of modern fashion in mature life, when the duties of wife and mother devolve upon them. The former is sprightly, active, and vivacious—her countenance lively, animated, and expressive of good health—her mind buoyant amidst all the troubles and vexations necessarily attendant upon the duties of a mother and a housewife: she enjoys the blessings of health, and the socciety of friends; she possesses a calm, serene mind, and is capable of meeting any reverse of fortune with fortitude—she is blessed with healthy offspring, and generally lives to see them grown up and acting for themselves. But the votary of modern fashion, how reversed her condition in after life! Her corporeal powers at an early period begin to evince decay—her intellectual faculties to manifest imbecility—her mind becomes irascible, and her temper fretful and peevish. Such women are generally sterile, or at most conceive but a few times. The first may possibly reach the full term of gestation, after which abortion is a common result. The functions of the generative organs finally become entirely or so nearly suspended as to render all possibility of a future offspring extremely doubtful.

III. *That constitutional debility is transmitted from the mother to the offspring, or at least, the effects wrought upon the constitution of the mother are felt by her offspring.*

The practice of excessive lacing interrupts to some extent the sympathetic communication between the uterus and mammæ, and when the functions of the latter are called into action, they are feebly and but imperfectly performed, consequently those unfortunate victims to the trammels of this delusive fashion, after the cares and duties of mothers devolve upon them, in many instances find it impossible to nourish at their breast their tender offspring. And besides the deprivation of that maternal pleasure, which all mothers find in nourishing their own offspring, she is constrained to witness as the consequence of artificial nourishment, the tortures and pains of disease, and frequently the agonies of death, from the supervention of colic, diarrhœa, or some of the many gastric and intestinal derangements to which children under such circumstances are peculiarly obnoxious. Here it is evident the sins of the mother are entailed upon her innocent offspring.

And farther, it is reasonable to suppose from the morbid condition of the system, met with in such women, that even when milk is secreted in sufficient quantity for nourishment, it is of a very deteriorated quality, calculated to ruin the health, and undermine the constitution of her child.

I have now answered, to the best of my humble abilities, the questions so often propounded to me; if satisfactorily, I shall be truly gratified—if otherwise, I hope a better explication will be furnished by some one better qualified than myself to do justice to so interesting and important a subject.—*Southern Medical and Surgical Journal.*

